# Supplementary material for: Implementation of a Patient Questionnaire in Community Pharmacies to Improve Care for Patients Using Combined Antithrombotic Therapy: A Qualitative Study
Source: Pharmacy (Basel). 2023 Apr 27;11(3):80. doi: 10.3390/pharmacy11030080 (PMC10204406; doi:10.3390/pharmacy11030080)
Supplement: Supplementary file 1 [file pharmacy-11-00080-s001.zip › pharmacy-2324088-supplementary.pdf]

pharmacy MDPI

Supplementary File S1. Antithrombotic questionnaire tool

Questionnaire for patients using combined antithrombotic therapy

Gender: *male / female / neutral* **Age:** …… year

According to our pharmacy information system you use two or more antithrombotic drugs

Part 1

Question 1: Which antithrombotics do you use?

	**Name antithrombotic**	**When did you start with these antithrombotics? (start date)**	**Do you know why you use these antithrombotics? (indication)**	**How long should you use these antithrombotics?**
Antithrombotic 1:		I don't know	Yes/No<br>If Yes, why*:	Rest of my life/<br>… weeks /<br>… months /<br>… year<br>I don't know
Antithrombotic 2:		I don't know	Yes/No<br>If Yes, why*:	Rest of my life/<br>… weeks /<br>… months /<br>… year<br>I don't know
Antithrombotic 3:		I don't know	Yes/No<br>If Yes, why*:	Rest of my life/<br>… weeks /<br>… months /<br>… year<br>I don't know

*If the answer is Yes, ask why the antithrombotic is used (indication) and ask when the most recent event was.

Notes:

Part 2

Question 2 Are you treated by a cardiologist, neurologist or a vascular surgeon?

	**Yes/No/ I don't know**	**If Yes In which hospital?**
A cardiologist (for your heart)		
A neurologist (for your brain)		
A vascular surgeon (for your blood vessels)		

Part 3

Question 3

**3a)** Have you ever experienced a stroke, or a TIA?	*Yes / no / don't know*<br><br>(If Yes, continue to question 3b, if no/don't know, continue to question 4)
**3b)** When have you experienced this (most recently)?	*Less than 3 weeks ago*<br><br>*More than 3 weeks ago*

Question 4

**4)** Have you ever experienced chest pain or heart cramps, which increased during exercise (Stable Angina Pectoris)? If yes, have you received medication for this?	*Yes / no / don't know*<br><br>*If yes, which drugs: (to rule out nitroglycerin)*

Question 5

**5a)** Have you ever had a heart attack (myocardial infarction)?<br>Or have you ever had a stent (tube) or operation (bypass) for your heart?<br><br>What kind of treatment did you receive? A tube in the heart (stent, PCI) or a bypass operation whereby there has been a detour of a blood vessel in the heart (CABG)?	*Yes / no / don't know*<br><br>(If yes, continue to question 5b.<br>If no / don't know, continue to question 6)
**5b)** When did this occur, most recently?	*Less than 1 month ago/*<br><br>*>1 month till 1 year ago/*<br><br>*1-2,5 years ago/*<br><br>*Longer than 2,5 years ago*

Question 6

**6a)** Have you had surgery on your heart valve?	*Yes / no / don't know*<br><br>(If yes, continue to question 6b.<br>If no / don't know, continue to question 7)
**6b)** Has your heart valve been replaced by surgery via a blood vessel in, for instance, a groin or shoulder (TAVI) or by an open-heart surgery with your chest open?	*TAVI / open heart surgery / don't know*<br><br>(If TAVI, continue to question 5e)
**6c)** Have you received a biological valve or a mechanical valve?	*Biological / mechanical / don't know*

**6d)** When did this occur, most recently?	*Shorter than 3 months /*<br>*3 to 6 months /*<br>*More than 6 months ago*

Question 7

**7a)** Has a doctor found narrowing of your vessels in your arms or legs (peripheral arterial disease) or in a vessel directing to the brain (carotid stenosis)?	*Yes / no / don't know*<br>(If yes, continue to question 7b.)
**7b)** Have you had blood vessel surgery for this?	*Yes / no / don't know*<br>(If yes, continue to question 7c.<br>(If no / don't know, continue to question 8.)
**7c)** Was this a surgery on varicose veins?	*Yes / no / don't know*
**7d)** Was a tube placed in the blood vessel (stent)?	*Yes / no / don't know*
**7e)** Has there been a bypass of a blood vessel?	*Yes / no / don't know*
**7f)** When did this occur, most recently?	*Shorter than 1 year /*<br>*1-2 years ago /*<br>*Longer than 2 years ago*

Question 8

**8)** Do you have, or have you ever had atrial fibrillation (atrial fibrillation / atrial flutter)	*Yes / no / don't know*

Question 9

**9)** Have you had a blood clot (thrombosis) in your lungs (pulmonary embolism) elsewhere, for example in your leg (deep vein thrombosis)?	*Yes / no / don't know*

Do you have any additional questions?

Notes:

Question for the conductor of the antithrombotic questionnaire tool

Do you think the information given by the patient is reliable? Yes/No/Some doubts

How did you conduct the antithrombotic questionnaire tool:
in person / by phone

Notes:

Supplementary File S2. Results assessment of correctness of combined antithrombotic therapy based on antithrombotic questionnaire tool

MBa and RvU individually assessed whether the combined antithrombotic therapy was correct or possibly incorrect based on the answers given by patients on indication, start date and intended duration. A patient should at least have answered the questions on indication and start date in order for the researchers to assess the therapy. In case the combined antithrombotic therapy did not match with the indication or when the duration of the therapy was beyond the expected duration the therapy was assessed as potentially incorrect. When the combined antithrombotic therapy did match with the indication and when duration of the therapy was within the expected duration the therapy was assessed as correct. When assessments differed, the therapy was discussed until consensus was reached. The general duration described in the European and Dutch guidelines were used to determine the expected duration of the antithrombotic therapy. The expected durations from these guidelines are shown/presented in Table 1 of this appendix.

Evaluation of combined antithrombotic therapy

We assessed the combined antithrombotic therapy of the patients who provided informed consent, and who knew the indication and the start date of the therapy. Of the 62 patients who provided informed consent, 56 patients (90%) knew indication(s) and the start date of the therapy. For these 56 patients therapy was assessed as potentially incorrect in 14 patients (25%). For these patients the community pharmacists were advised to contact the physician to check whether the multiple antithrombotic therapy was still indicated. For the other patients expected stop dates were provided by the research team.

Supplementary File S2 Table 1 General duration of multiple antithrombotic therapy

**Indication**	**Duration of therapy**	**Guideline**
**Dual antiplatelet therapy (DAPT)**		
Minor noncardioembolic ischemic stroke < 21 days ago, who did not receive IV alteplase	Maximum of 21 days	2019 FMS Herseninfarct en hersenbloeding (Cerebral infarction and cerebral haemorrhage)
Acute coronary syndrome	In general 12 months*	2017 ESC focused update on dual antiplatelet therapy in coronary artery disease
PCI in stable CAD setting	In general 6 months†	2017 ESC focused update on dual antiplatelet therapy in coronary artery disease
TAVI (without high bleeding risk)	In general 3 months‡	2017 ESC/EACTS Guidelines for the management of valvular heart disease

Revascularization percutaneous in patients with lower extremity artery disease<br><br>Infra-inguinal stent implementation	At least 1 month, in research setting till 12 months	2017 ESVS/ESC Guidelines on the diagnosis and treatment of peripheral arterial diseases
**Double therapy (DAT)**		
Indication for therapeutic anticoagulation and ACS < 1 year	12 months after ACS ¶/§	2017 ESC focused update on dual antiplatelet therapy in coronary artery disease
Indication for therapeutic anticoagulation and PCI < 1 year	6-12 months after PCI ¶/§	2017 ESC focused update on dual antiplatelet therapy in coronary artery disease<br>2018 ESC/EACTS Guidelines on myocardial revascularization
Indication for therapeutic anticoagulation and TAVI procedure < 6 months ago	6 months after TAVI ¶/§	2017 ESC/EACTS Guidelines for the management of valvular heart disease
Indication for therapeutic anticoagulation and endovascular revascularization (stent/graft)	At least 1 month	2017 ESVS/ESC Guidelines on the diagnosis and treatment of peripheral arterial diseases
**Triple therapy (TAT)**		
Indication for therapeutic anticoagulation and ASC for which PCI < 1 month	1 month after PCI	2017 ESC focused update on dual antiplatelet therapy in coronary artery disease
Atrial fibrillation, NSTEMI and PCI < 1 week	1 week after PCI ¶	2020 ESC Guidelines for the management of<br>acute coronary syndromes in patients<br>presenting without persistent ST-segment<br>elevation

DAPT= dual antiplatelet therapy
PCI= Percutaneous coronary intervention
TAVI= Transcatheter aortic valve implantation
CAD= Coronary artery disease
ACS = Acute coronary syndrome

STEMI= ST segment elevation myocardial infarction

* 3- 36 months (depending on risk factors, for instance PRECISE-DAPT and DAPT score)

† 1-30 months (depending on risk factors, for instance PRECISE-DAPT and DAPT score)

‡3-6 months

¶ treatment can be extended in patients with high ischaemic risk and in patients with mechanical prosthesis and atherosclerotic disease

§Shorter duration may be considered in patients with a high bleeding risk.

Supplementary File S3. Interview guide pharmacist

General:

1. You have used the questionnaire for a couple of weeks now, could you tell me your experience with this questionnaire?
2. You have told us previously that you intended to use the questionnaire {by phone/at the counter}, did you use it as you intended? [Fidelity, Process, planning]
   - If not, could you tell me why u used another approach?
3. Did you change anything in the questionnaire? (for instance, asked less questions?) [Intervention Characteristics + Fidelity]
4. How complicated was it to use the questionnaire? [Intervention Characteristics, Complexity]
   - How much time does it take to use the questionnaire?
   - Do you think this time is acceptable?
5. How well does the questionnaire fit in your daily pharmacy practice? [Implementation Climate, Compatibility]
6. How many patients have you contacted and how many patients participated?
7. Which reactions did you receive from patients? [Outersetting, Patients Needs & Resources]

Experiences & Opinion:

8. What was your motivation to participate in this study?
9. Are there other healthcare related projects your pharmacy participates in? [Inner Setting, Culture]
10. How do you pay attention to patients who use combined antithrombotic drugs? [Intervention Characteristics, Relative Advantage]
11. In which way could this questionnaire be an addition to the current process of patients who use combined antithrombotic drugs? [Implementation Climate, Compatibility]
12. Do you see added value in using the questionnaire? [Characteristics of Individuals, Knowledge & Beliefs about the Intervention]
    - Yes, in which way?
    - No, why not?
13. How will the use of the questionnaire affect the contact with a physician? [Outer Setting, Cosmopolitanism]
14. How well does the questionnaire fit with your norms and values and the norms and values within the organization? [Implementation Climate, Compatibility]
15. What could be reasons for you, for not using the questionnaire? [Implementation Climate, Relative Priority]
16. Do you believe you have enough information to analyze the questionnaire? [Readiness for Implementation, Access to Knowledge & Information]
    - How many interventions have you performed?
17. Do you believe you are able to use the questionnaire? [Characteristics of Individuals, Self-Efficacy]
18. For how many of your patients (who use combined antithrombotic therapy) do you think you can use this questionnaire? (%, For instance, based on health literacy skills of the patients)
19. What kind of changes or alterations are necessary to the questionnaire so it can work effectively in your setting? [Intervention Characteristics, Adaptability]
20. Would you like to continue using the questionnaire in daily practice?? [Process, Reflecting & Evaluating]

- Yes, how do you want to use the questionnaire (in the ideal situation)?
- No, why not?

Supplementary File S4. Interview guide pharmacy technician

General:

1. You have used the questionnaire for a couple of weeks now, could you tell me your experience with this questionnaire?
2. You have told us previously that you intended to use the questionnaire {by phone/at the counter}, did you use it as you intended? [Fidelity, Process, planning]
    - If not, could you tell me why u used another approach?
3. Did you change anything in the questionnaire? (for instance, asked less questions?) [Intervention Characteristics + Fidelity]
4. How complicated was it to use the questionnaire? [Intervention Characteristics, Complexity]
    - How much time does it take to use the questionnaire?
5. How well does the questionnaire fit in your daily pharmacy practice? [Implementation Climate, Compatibility]
6. How many patients have you contacted and how many patients participated?
7. Which reactions did you receive from patients? [Outersetting, Patients Needs & Resources]

Experiences & Opinion:

8. Are there other healthcare related projects your pharmacy participates in? [Inner Setting, Culture]
9. How do you pay attention to patients who use combined antithrombotic drugs? [Intervention Characteristics, Relative Advantage]
10. What kind of support did you receive from your pharmacist before the use of the questionnaire? For instance, explanation about why the questionnaire is used and how the questionnaire should be used? [Readiness for Implementation, Leadership Engagement]
11. In what way did you receive sufficient time and space to use the questionnaire? [Readiness for Implementation, Available Resources]
12. Who would you ask if you had questions about the use of the questionnaire? [Readiness for Implementation, Access to Knowledge & Information]
13. Do you believe you are able to use the questionnaire? [Characteristics of Individuals, Self-Efficacy]
14. What could be reasons for you, for not using the questionnaire? [Implementation Climate, Relative Priority]
15. Do you think that your colleagues could use this questionnaire? [Characteristics of Individuals, Self-Efficacy]
    - No, why not?
16. What kind of changes or alterations are necessary to the questionnaire so it can work effectively in your setting? [Intervention Characteristics, Adaptability]
17. Would you like to continue using the questionnaire in daily practice? [Process, Reflecting & Evaluating]
    - Yes, how do you want to use the questionnaire (in the ideal situation)?
    - No, why not?

Supplementary File S5. Adapted antithrombotic questionnaire tool

Questionnaire for patients using combined antithrombotic therapy

Gender: *male / female / neutral* **Age:** …… year

Name patient: ………………………………..

Date of birth: ………………………………….

According to our pharmacy information system you use two or more antithrombotic drugs

Part 1

Question 1: Which antithrombotics do you use?

	Name antithrombotic	When did you start with these antithrombotics? (start date)	Do you know why you use these antithrombotics? (indication)	How long should you use these antithrombotics?
Antithrombotic 1:		I don't know	Yes/No<br><br>If Yes, why*:	Rest of my life/<br>… weeks /<br>… months /<br>… year<br><br>I don't know
Antithrombotic 2:		I don't know	Yes/No<br><br>If Yes, why*:	Rest of my life/<br>… weeks /<br>… months /<br>… year<br><br>I don't know
Antithrombotic 3:		I don't know	Yes/No<br><br>If Yes, why*:	Rest of my life/<br>… weeks /<br>… months /<br>… year<br><br>I don't know

*If the answer is Yes, ask why the antithrombotic is used (indication) and ask when the most recent event of the disease was.

Notes:

Part 2

Question 2 Are you treated by a cardiologist, neurologist or a vascular surgeon?

	Yes/No/<br>I don't know	If Yes<br>In which hospital?
A cardiologist (for your heart)		
A neurologist (for your brain)		
A vascular surgeon (for your blood vessels)		

Part 3

Question 3

**3a)** Have you ever experienced a stroke, or a TIA?	*Yes / no / don't know*<br><br>(If Yes, continue to question 3b, if no/don't know, continue to question 4)
**3b)** When have you experienced this (most recently)?	*Less than 3 weeks ago*<br><br>*More than 3 weeks ago*

Question 4

**4)** Have you ever experienced chest pain or heart cramps, which increased during exercise (Stable Angina Pectoris)? If yes, have you received medication for this?	*Yes / no / don't know*<br><br>*If yes, which drugs: (to rule out nitroglycerin)*<br><br>*Continue to question 5*

Question 5

**5a)** Have you ever had a heart attack (myocardial infarction)?<br>Or have you ever had a stent (tube) or operation (bypass) for your heart?<br><br>What kind of treatment did you receive? A tube in the heart (stent, PCI) or a bypass operation whereby there has been a detour of a blood vessel in the heart (CABG)?	*Yes / no / don't know*<br><br>(If yes, continue to question 5b.<br>If no / don't know, continue to question 6)
**5b)** When did this occur, most recently?	*Less than 1 month ago/*<br><br>*>1 month till 1 year ago/*<br><br>*1-2,5 years ago/*<br><br>*Longer than 2,5 years ago*

Question 6

**6a)** Have you had surgery on your heart valve?	*Yes / no / don't know*

	(If yes, continue to question 6b.<br>If no / don't know, continue to question 7)
**6b)** Has your heart valve been replaced by surgery via a blood vessel in, for instance, a groin or shoulder (TAVI) or by an open-heart surgery with your chest open?	*TAVI / open heart surgery / don't know*<br><br>(If TAVI, continue to question 5e)
**6c)** Have you received a biological valve or a mechanical valve?	*Biological / mechanical / don't know*
**6d)** When did this occur, most recently?	*Shorter than 3 months /*<br><br>*3 to 6 months /*<br><br>*More than 6 months ago*

Question 7

**7a)** Has a doctor found narrowing of your vessels in your arms or legs (peripheral arterial disease) or in a vessel directing to the brain (carotid stenosis)?	*Yes / no / don't know*<br><br>(If yes, continue to question 7b.)
**7b)** Have you had blood vessel surgery for this?	*Yes / no / don't know*<br><br>(If yes, continue to question 7c.<br>(If no / don't know, continue to question 8.)
**7c)** Was this a surgery on varicose veins?	*Yes / no / don't know*
**7d)** Was a tube placed in the blood vessel (stent)?	*Yes / no / don't know*
**7e)** Has there been a bypass of a blood vessel?	*Yes / no / don't know*
**7f)** When did this occur, most recently?	*Shorter than 1 year /*<br><br>*1-2 years ago /*<br><br>*Longer than 2 years ago*

Question 8

**8)** Do you have, or have you ever had atrial fibrillation (atrial fibrillation / atrial flutter)	*Yes / no / don't know*

Question 9

**9)** Have you had a blood clot (thrombosis) in your lungs (pulmonary embolism) elsewhere, for example in your leg (deep vein thrombosis)?	*Yes / no / don't know*

Do you have any additional questions?

Notes:

Supplementary File S6. Additional Quotes

(sub)Construct	Quote
Complexity	*"When you read the questionnaire well before you use it then it is not complex to use" (Pharmacy technician, female 24 years old)*
Cosmopolitism	*We had 3 similar quotes from pharmacy staff on this construct.*<br>*"If you already know the indication and you can estimate the expected duration than you can call the physician more prepared."*
Compatibility	*" You do need to take the time for the questionnaire, you can't just do it in between jobs or on the counter of the pharmacy, therefore the conversation [use of questionnaire] is too long in a busy pharmacy" (pharmacy technicians, female age 24 and age 27)*
Knowledge and beliefs about the intervention	*"Yes, most of the patients know quite something (about their anticoagulation therapy) that was remarkable to me. So that was good to see. And then you see the added value of the questionnaire and that you can use it quite well in daily practice so you can better estimate what kind of anticoagulation someone needs. So I really liked using it [the questionnaire]." (Pharmacist, female, 27 years-old)*
Relative priority	*" Time pressure could be a barrier yes, when there is a line of patients that are waiting then you do not use the questionnaire. To use the questionnaire, you need to take the patients apart or make an appointment and in the normal workflow sometimes things can be forgotten" (Pharmacist, female, 56 years old)*
Self-efficacy	*"I believe that pharmacy technicians could use this questionnaire when clearly is explained to them for which indications two or more antithrombotics are indicated. Then I believe that pharmacy technicians could use this questionnaire." (Pharmacy technician, female, 35 years old)*
